# Photobiomodulation Therapy Associated with Heterologous Fibrin Biopolymer and Bovine Bone Matrix Helps to Reconstruct Long Bones

**DOI:** 10.3390/biom10030383

**Published:** 2020-03-02

**Authors:** Marcelie Priscila de Oliveira Rosso, Aline Tiemi Oyadomari, Karina Torres Pomini, Bruna Botteon Della Coletta, João Vitor Tadashi Cosin Shindo, Rui Seabra Ferreira Júnior, Benedito Barraviera, Claudia Vilalva Cassaro, Daniela Vieira Buchaim, Daniel de Bortoli Teixeira, Sandra Maria Barbalho, Murilo Priori Alcalde, Marco Antonio Hungaro Duarte, Jesus Carlos Andreo, Rogério Leone Buchaim

**Affiliations:** 1 Department of Biological Sciences (Anatomy), Bauru School of Dentistry, University of São Paulo (USP), Alameda Dr. Octávio Pinheiro Brisolla, 9-75-Vila Universitaria, Bauru 17012-901, São Paulo, Brazil; marcelierosso@usp.br (M.P.d.O.R.); lilitiemi@hotmail.com (A.T.O.); karinatorrespomini@gmail.com (K.T.P.); brunacoletta@usp.br (B.B.D.C.); jvshindo@gmail.com (J.V.T.C.S.); jcandreo@usp.br (J.C.A.); 2 Center for the Study of Venoms and Venomous Animals (CEVAP), São Paulo State University (Univ. Estadual Paulista, UNESP), Botucatu 18610-307, São Paulo, Brazil or rseabra@cevap.unesp.br (R.S.F.J.); bbviera@gnosis.com.br (B.B.); claudia.v.cassaro@gmail.com (C.V.C.); 3 Postgraduate Program in Structural and Functional Interactions in Rehabilitation, University of Marilia (UNIMAR), Avenue Hygino Muzzy Filho, 1001, Marília 17525-902, São Paulo, Brazil; danibuchaim@usp.br (D.V.B.); daniel.dbt@hotmail.com (D.d.B.T.); smbarbalho@gmail.com (S.M.B.); 4 Medical School, University Center of Adamantina (UniFAI), Nove de Julho Street, 730-Centro, Adamantina 17800-000, São Paulo, Brazil; 5 Department of Health Science, University of the Sacred Heart (USC), Bauru 17011-160, São Paulo, Brazil; murilo.alcalde@usp.br; 6 Department of Dentistry, Endodontics and Dental Materials, Bauru School of Dentistry, University of São Paulo (USP), Bauru 17012-901, São Paulo, Brazil; mhungaro@fob.usp.br

**Keywords:** biomaterials, bone regeneration, fibrin biopolymer, low-level laser therapy, photobiomodulation therapy

## Abstract

Bone defects cause aesthetic and functional changes that affect the social, economic and especially the emotional life of human beings. This complication stimulates the scientific community to investigate strategies aimed at improving bone reconstruction processes using complementary therapies. Photobiomodulation therapy (PBMT) and the use of new biomaterials, including heterologous fibrin biopolymer (HFB), are included in this challenge. The objective of the present study was to evaluate the influence of photobiomodulation therapy on bone tibial reconstruction of rats with biomaterial consisting of lyophilized bovine bone matrix (BM) associated or not with heterologous fibrin biopolymer. Thirty male rats were randomly separated into three groups of 10 animals. In all animals, after the anesthetic procedure, a noncritical tibial defect of 2 mm was performed. The groups received the following treatments: Group 1: BM + PBMT, Group 2: BM + HFB and Group 3: BM + HFB + PBMT. The animals from Groups 1 and 3 were submitted to PBMT in the immediate postoperative period and every 48 h until the day of euthanasia that occurred at 14 and 42 days. Analyses by computed microtomography (µCT) and histomorphometry showed statistical difference in the percentage of bone formation between Groups 3 (BM + HB + PBMT) and 2 (BM + HFB) (26.4% ± 1.03% and 20.0% ± 1.87%, respectively) at 14 days and at 42 days (38.2% ± 1.59% and 31.6% ± 1.33%, respectively), and at 42 days there was presence of bone with mature characteristics and organized connective tissue. The µCT demonstrated BM particles filling the defect and the deposition of new bone in the superficial region, especially in the ruptured cortical. It was concluded that the association of PBMT with HFB and BM has the potential to assist in the process of reconstructing bone defects in the tibia of rats.

## 1. Introduction

Traumas, congenital anomalies and surgeries are morbid conditions that can lead to transient or permanent bone defects, often subject to reconstruction. These conditions influence the patient’s life, affecting the social, psychic, aesthetic and work spheres with consequent increase of the financial costs to the health system [1,2].

Preclinical and clinical research has already shown that autogenous bone grafting (ABG) is the standard material for bone grafting in larger defects, which do not repair completely spontaneously. However, the limitations of supply, involvement of two surgical areas and morbidity mainly related to the collection of the autograft can be considered disadvantages of this technique. In this way, several bone substitutes are tested and used in surgeries, both dental (on facial bones) and orthopedic (on long bones) [3].

Biomaterials used as bone substitutes must be biocompatible, biodegradable and also form a scaffold for osteoconductivity, in addition to having porosity similar to the natural bone of the recipient bed and allowing the growth of osteoinductivity factors. Among the products available commercially in dentistry and orthopedic medicine, Geistlich Bio-Oss^®^ is noteworthy due to the several published scientific works and wide use in the world [4,5].

In addition, researchers are looking for an ideal scaffold to be used in conjunction with bovine bone matrix (BM). In this context, fibrin sealant stands out as an aid in granule adhesion, in the formation of a stable fibrin network and in the maintenance of the medium as a support for cell growth [6,7,8,9]. Commercial sealants are all homologous, that is, made from human blood components, which makes them extremely expensive due to the scarcity of raw materials for production. To overcome these challenges, the Center for the Study of Venoms and Venomous Animals (CEVAP) from São Paulo State University (UNESP), Sao Paulo, Brazil, has been studying and developing heterologous fibrin biopolymer (HFB) from animal origin materials since the 1990s. This heterologous biomaterial is biocompatible, having hemostatic, adhesive, sealant, scaffold and drug delivery properties [9,10,11]. This bioproduct is being successfully tested in reconstruction processes when applied to various tissues [6,12,13,14,15].

In addition to specific treatments, rehabilitation therapy by techniques complementary to therapeutic treatment has been increasingly applied, especially photobiomodulation therapy (PBMT), with the use of low intensity laser [8,13,16,17,18]. Its photon energy reaches the cell nucleus increasing the synthesis of deoxyribonucleic acid (DNA) and ribonucleic acid (RNA) and consequent protein synthesis [19]. Furthermore, it increases the adenosine triphosphate (ATP) and cellular mitotic activity [20]. Defined as the application of energy directly from noncoherent (light emitting diode) or coherent (lasers) light, with varying wavelengths between 405 and 1100 nm, it is capable of producing photochemical effects by modulating cells [21,22]. This new technology is the current focus of studies of the scientific community for presenting reparative mechanisms of action. However, there is no consensus on its effective parameters, because if parameters are not properly delineated, they do not have beneficial effects on tissue regeneration, making this a current focus of research [19,21,23,24,25].

Considering the absence of previous studies that have used the unique heterologous fibrin biopolymer derived from snake venom in the world, (HFB) [9], associated with bone substitute of proven grafting quality [26,27], with results similar to the gold standard (ABG) [28], it was decided to conduct this research in noncritical defects of rat tibia in order to evaluate the therapeutic potential of the combination of these products, associating a recent PBMT protocol, already demonstrated in the literature as promising to treat bone defects [8,16], but not yet explored along the lines of this research.

In view of the problem and the objective previously presented, the hypothesis of the present study is that the combination of photobiomodulation therapy with bone substitutes improves the repair process of bone defects.

## 2. Materials and Methods

### 2.1. Animal Maintenance

All experimental procedures are in accordance with the Ethical Principles on Animal Experimentation adopted by the Brazilian College of Animal Experimentation (COBEA), and this project was approved under No. 006/2019.

Thirty adult male Wistar rats (*Rattus norvegicus*) were used (90 days), weighing an average of 470 g, provided by the Central Biottery of the Ribeirão Preto School of Dentistry, University of São Paulo (FORP-USP). The animals were kept in an animal house in appropriate cages, four animals per box, receiving water and food “ad libitum,” without restrictions on movement, respecting 12-h light cycles, acclimatized by air extractors and air conditioning.

### 2.2. Experimental Design

All animals underwent experimental surgery, with defect filled with freeze-dried bovine bone matrix (BM) biomaterial, and were randomized into three groups:

Group 1: Lyophilized bovine bone matrix + photobiomodulation therapy (BM + PBMT), *n* = 10 (biomaterial and biostimulated);

Group 2: Lyophilized bovine bone matrix + heterologous fibrin biopolymer (BM + HFB), *n* = 10 (biocomplex composed of biomaterial mixed to heterologous fibrin biopolymer);

Group 3: Lyophilized bovine bone matrix + heterologous fibrin biopolymer + photobiomodulation therapy (BM + HFB + PBMT), *n* = 10 (biocomplex composed of biomaterial mixed to heterologous fibrin biopolymer and biostimulated).

### 2.3. Lyophilized Bovine Bone Matrix (BM)

The bone matrix biomaterial used in the present research was bovine demineralized bone, commercially called Bio-Oss^®^ (Geistlich Pharma AG, Wolhusen, Switzerland; Ministry of Health Registration No. 806.969.30002). It is characterized as porous bovine bone matrix with particle size between 0.25–1 mm, packed in 2 g flasks with corresponding porosity of 75–80% of the total volume between 10 nm to 100 µm [29].

### 2.4. Heterologous Fibrin Biopolymer (HFB)

In the Group 2 (BM + HFB) and 3 (BM + HFB + PBMT) animals, the biomaterial was mixed with the heterologous fibrin biopolymer forming a biocomplex to act as drug delivery. This mixture formed a biological framework for the biomaterial particles, favoring their introduction into the defect and at the same time maintaining them in the surgical bed. The HFB used in the research was kindly provided by CEVAP itself and had in its composition three vials: one vial with fraction 1 (thrombin-like), one vial with fraction 2 (fibrinogen-rich cryoprecipitate obtained from buffalo blood) and one vial of diluent (calcium chloride). This product is under patent under Registration Numbers BR1020140114327 and BR1020140114360.

The HFB was handled according to the standardization proposed by Ferreira Jr. et al. [10], which describes a 1:2:1 ratio as follows: one part fraction 1 (thrombin-like), two parts fraction 2 (cryoprecipitate rich in fibrinogen) and one part diluent (fraction 3, calcium chloride), with an amount of microliters according to the size of the defect to be filled. The components of the HFB remained frozen until the moment of use, when they were thawed and mixed in the proportions previously established to generate a stable clot according to the following protocol: fraction 1 = 10 μL, fraction 2 = 20 μL and diluent = 10 μL. Fractions were dosed using Gibson^®^ micropipettes with disposable tips. The application sequence was as follows: fraction 1 was placed over the BM in an eppendorf, followed by mixing fraction 2 with the diluent in another Eppendorf^®^, where they were homogenized [30] and then applied to the BM, forming a biocomplex that was inserted into the defect.

### 2.5. Monocortical Defect Surgery

All surgical procedures were performed at the Department of Biological Sciences at the Anatomy Mesoscopy Laboratory of the Bauru School of Dentistry, University of São Paulo, Brazil, by the same team of researchers.

For the experimental surgery, the rats were submitted to general anesthesia with intramuscular injection of ketamine (0.3 mL/kg) (Dopalen^®^, Ceva, Paulinia, São Paulo, Brazil) and xilasine (0.3 mL/kg) (Anasedan^®^, Ceva, Paulínia, São Paulo, Brazil), and trichotomy was performed in the lateral dorsal region of the left pelvic after with disinfection of the operative field by 10% povidone–iodine (PI) topical solution. Next, with a No. 15 scalpel blade, a 20 mm long linear incision was made in the craniocaudal direction, sectioning the skin and muscle fasciae, reaching the periosteum and extending it for tibial exposure. With an AR 6 steel carbide drill (Beavers Dental^®^, Morrisburg, ON, Canada, K0C 1 × 0/Ormex SA) coupled to a 1500 rpm low-speed micromotor, a 2 mm diameter cavity was prepared [31,32,33,34,35] in a depth reaching the bone marrow without damaging the contralateral cortical [18,36,37,38,39], with abundant irrigation of 0.9% sodium chloride solution.

For each animal in the three groups, the cavity was filled by BM in the amount of 0.012 g (defined in a preliminary pilot study), which was weighed on an analytical balance (Micronal^®^, Precision Equipment, São Paulo, Brazil). In Group 1 (BM + PBMT), after weighing the biomaterial, enough saline was added to adhere the granules and facilitate deposition inside, completing the entire defect. In Groups 2 (BM + HFB) and 3 (BM + HFB + PBMT), the heterologous fibrin biopolymer was used as a scaffold for bone matrix, forming a biocomplex.

**Figure 1:**
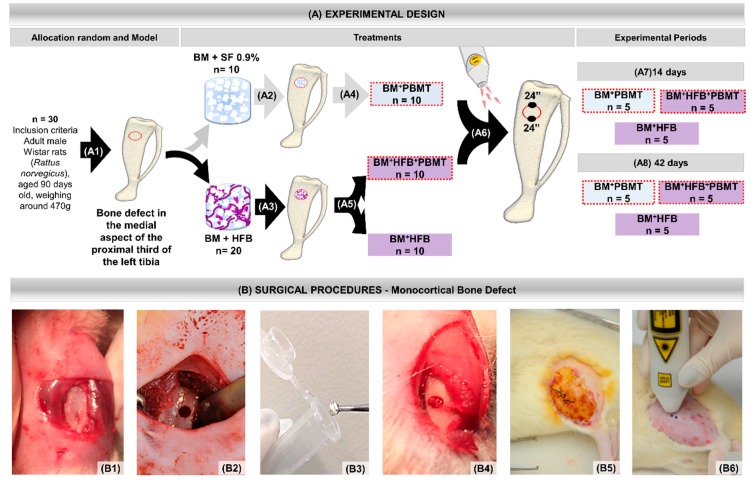
(**A**) Experimental design. (**A1**) Monocortical osteotomy in the medial aspect of the proximal third of the tibia. (**A2**) Defects filled with biomaterial (BM) mixed with saline (SF). (**A3**) Defects filled with biomaterial—lyophilized bovine bone matrix (BM) mixed with heterologous fibrin biopolymer (HFB). (**A4**) Group 1 (BM *+* PBMT): defects filled with biomaterial and photobiomodulation therapy (PBMT). (**A5**) Group 3 (BM *+* HFB *+* PBMT): defects filled with biomaterial mixed with heterologous fibrin biopolymer and photobiomodulation therapy. Group 2 (BM *+* HFB): defects filled with biomaterial and nonlaser biostimulated. (**A6**) Illustration of the two laser-irradiated points for 24 s each. (**A7**,**A8**) Euthanasia periods of 14 and 42 days: five animals from each group/period. (**B**) Surgical procedures—cortical defect bone: (**B1**) medial aspect of the proximal third of the left tibia; (**B2**) monocortical bone defect of 2 mm; (**B3**) biomaterial mixed with fibrin biopolymer; (**B4**) defects filled with biomaterial mixed with fibrin biopolymer in the surgical cavity; (**B5**) tegument suture with 4–0 silk thread. (**B6**) Schematic representation of laser application.

After filling the cavities, the periosteum and other tissues of the operated region were repositioned and sutured using 4–0 Ethicon^®^ silk thread (Ethicon^®^, Johnson & Johnson Company, New Orleans, LA, USA). The operative acts were always performed by a single operator subjecting the animals to the same conditions, and the experimental design is detailed in Figure 1.

Immediately after the surgical procedures, the animals received paracetamol analgesic (Paracetamol^®^, Medley, São Paulo, Brazil) at a dose of 200 mg/kg, dissolved in the water available in the drinker for 3 days.

### 2.6. Photobiomodulation Therapy

**Figure 2:**
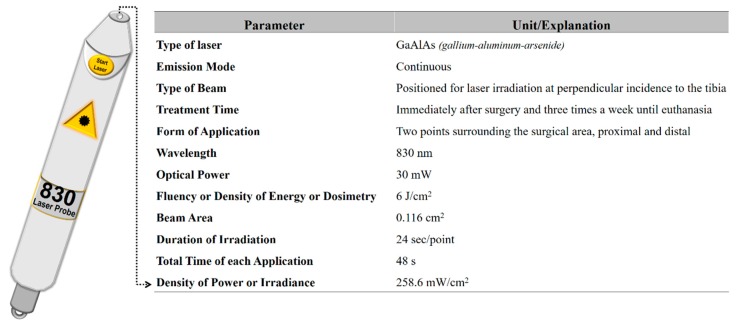
Details of the parameters used for PBMT application.

For animals from Groups 1 (BM + PBMT) and 3 (BM + HFB + PBMT), the GaAlAs (gallium–aluminum–arsenide) laser (Laserpulse IBRAMED^®^, Amparo, São Paulo, Brazil) was applied. The design of the parameters is described in Figure 2. In all applications, the laser beam emissions were calibrated on the device itself and previously tested to certify the dose [8,16].

### 2.7. Collection of Samples and Histological Procedures

After 14 and 42 days after surgery, five animals from each group, per period, underwent general anesthesia with an intramuscular injection of ketamine and xilazine mixture. The samples were fixed in 10% buffered formaldehyde for a period of 72 h and then computed microtomographically. The next step was decalcification in 10% ethylenediaminetetraacetic acid (EDTA) solution containing 4.13% Titriplex^®^ III (Merck KGaA, Darmstadt, Germany) and 0.44% sodium hydroxide (Labsynth, São Paulo, Brazil) and given sequence in standardized histological processing [8,16]. Subsequently, longitudinal, semiserial sections (50 µm interval) of defects of 5 µm thickness and stained with hematoxylin–eosin were performed.

### 2.8. X-ray Computed Microtomography Analysis (µ-CT)

The samples were placed in a cylindrical acrylic tube and allocated inside the SkyScan 1174v2 microtomograph (μ-CT Bruker microCT^®^, Kontich, Belgium), obtaining images with 13.76 µm voxel, 0.73° per sequence. Next, the two-dimensional reconstruction and realignment analyses were performed using the NRecon^®^ 1.6.9 and DataViewer^®^ 1.4.4.0 software, respectively. For the reconstitution of the three-dimensional images, the CTVox^®^ 2.4.0 r870 software (Bruker microCT) was used.

### 2.9. Histomorphometric and Histological Analysis

Images were obtained by Olympus^®^ BX50 light microscope (Olympus^®^ Corporation, Tokyo, Japan) on 4×, 40× and 100× lenses at the FOB-USP Anatomy Laboratory using the DP Controller software 3.2.1.276-2001-2006 (Olympus^®^ Corporation, Tokyo, Japan).

For the histomorphological description of the bone defect areas, 40× and 100× images were used in all specimens, considering the entire extent of the defect in order to analyze granulation tissue, inflammatory infiltrate, presence and quality (immature or mature/lamellar bone) and the degree of filling of the newly formed tissue, regarding the interaction between the HFB and PBMT with the BM used.

Quantitative analysis about the percentage of new bone volume was evaluated by 4× images using the point count planimetry method. For this, a previously established grid with 88 points [34,40] was superimposed on the histological section image of each animal, each point that overlapped the newly formed tissue was considered, and the total density was evaluated by the occupation in % of the image covering the defect in its entirety.

The grid size used was 13.2 × 9.6 cm with 1.2 cm spacing between each point marked on a transparent sheet. The measurement of the area densities of the analyzed sections was performed using the equation: D = ΣPN / PT × 100, where PN indicates the number of overlapping points in new bone formation and PT the number of total points included in the overlapping grid. The percentage value was related to the average of all animals analyzed (Figure S1).

### 2.10. Statistical Analysis

Data were submitted to analysis of variance (ANOVA) to detect possible differences between groups. The ANOVA assumptions, residual normality and variance homogeneity, were verified, respectively, by the Shapiro–Wilk and Bartlett tests, both at 5% probability. Subsequently, the means were compared by the Tukey’s test at 5% probability. Within each treatment, the comparison of new bone formation as a function of the treatment period (14 and 42 days) was assessed by the Student’s *t*-test at 5% probability. All analyses were conducted using the R software (R Core Team^®^, 2019, The R Foundation for Statistical Computing, Vienna, Austria).

## 3. Results

### 3.1. General Evaluation

No complications were seen in the postoperative period of the animals, with normal healing and no signs of infection. Signs of pain-related behavioral changes such as decreased movement or weight loss were also not evident.

### 3.2. Microtomographic Evaluation

**Figure 3:**
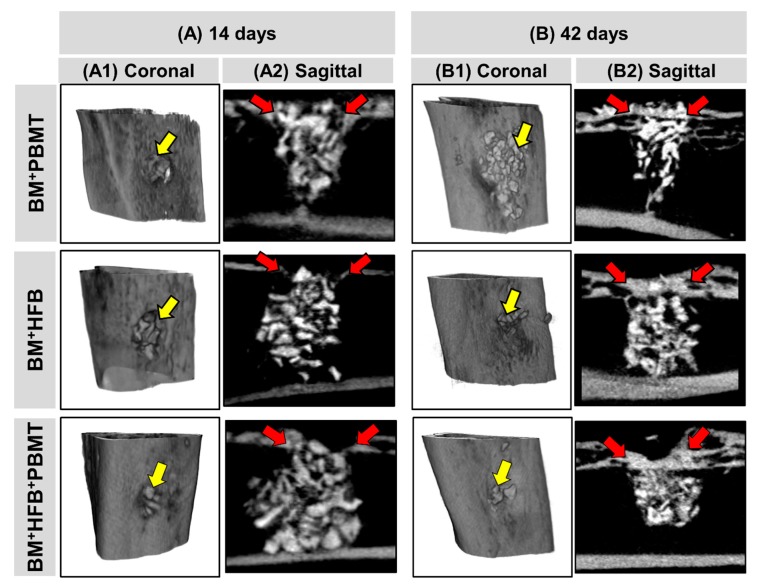
Representative microcomputed tomography (µCT) image of the proximal third of the tibia for each rat group: 1 (BM + PBMT) (lyophilized bovine bone matrix with photobiomodulation therapy), 2 (BM + HFB) (lyophilized bovine bone matrix plus heterologous fibrin biopolymer) and 3 (BM + HFB + PBMT) (lyophilized bovine bone matrix plus heterologous fibrin biopolymer with photobiomodulation therapy) in the periods of (**A**) 14 and (**B**) 42 days. (**A1**,**B1**) Three-dimensional coronal section shows the cortical region of the defect, biomaterial particles (yellow arrow). (**A2**,**B2**) Two-dimensional sagittal section shows the cortical and medullary region of the defect filled by BM, defect in the cortical bone (red arrows).

The images analyzed by µCT observed in Figure 3 showed that the biomaterial resembles the cortical bone, mainly in its radiopacity, not allowing the quantitative verification of the newly formed bone tissue.

At 14 days (Figure 3(A1)), it is possible to notice the monocortical bone defect with definition of its borders and covered with biomaterial in all groups. In Group 2 (BM + HFB), the particles appear closer to each other, and in Groups 1 (BM + PBMT) and 3 (BM + HFB + PBMT) there was a tendency for bone formation in the ruptured cortical (Figure 3(A2)).

At 42 days (Figure 3(B1)), visually, there is new bone covering the cortical area, with particles of the biomaterial in the midst of this new formation with bone healing process in continuity. There was a visual difference in bone board thickness in relation to the days, being the thickest in this period. In Group 1 (BM + PBMT), some particles of the biomaterial exceeded the defect limits (Figure 3(B1)); in Groups 2 (BM + HFB) and 3 (BM + HFB + PBMT), the defect site appears to be thicker bone undergoing remodeling.

### 3.3. Histological Evaluation

**Figure 4:**
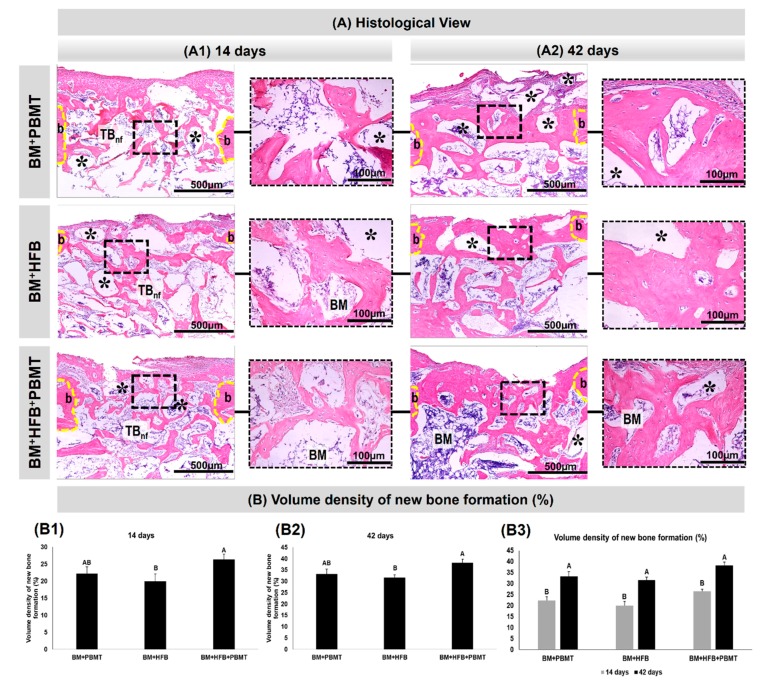
(**A**) Histological views at 14 and 42 days in tibia defects filled with lyophilized bovine bone matrix graft with photobiomodulation therapy (Group 1, BM + PBMT), lyophilized bovine bone matrix graft plus heterologous fibrin biopolymer (Group 2, BM + HFB) and lyophilized bovine bone matrix graft plus heterologous fibrin biopolymer with photobiomodulation therapy (Group 3, BM + HFB + PBMT). Newly formed trabecular bone (TBnf), bone graft particles (asterisk), defect border (b), bone marrow (BM). (**B**) Graphs of volume density. Graphs (**B1**,**B2**) demonstrate the comparisons of the volume density of the new bone formed between the groups studied in the same period of experimentation (14 or 42 days). In (**B3**), the volume density of new bone formed in the same group in the two experiment periods (14 or 42 days) is compared *(n* = 5/group). Different uppercase letters (A ≠ B) indicate a statistically significant difference (*p* < 0.05). (hematoxylin and eosin (HE); original 10× magnification, bar = 500 µm; 40× magnified images, bar = 100 µm).

**Figure 5:**
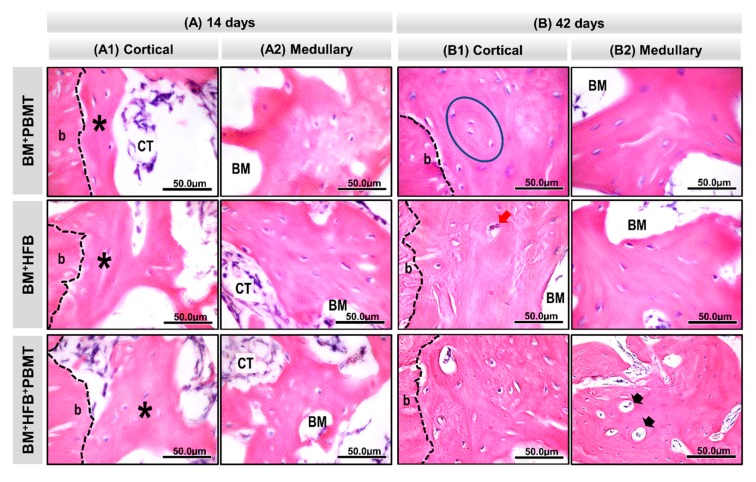
Time course of monocortical defect healing in rats tibia in groups: Group 1 (BM + PBMT) (defects filled with lyophilized bovine bone matrix (xenograft) with photobiomodulation therapy), Group 2 (BM + HFB) (defects filled with lyophilized bovine bone matrix plus heterologous fibrin biopolymer) and Group 3 (BM + HFB + PBMT) (defects filled with lyophilized bovine bone matrix plus heterologous fibrin biopolymer with photobiomodulation therapy) in the periods of 14 and 42 days. (**A**) At 14 days. (**A1**) In cortical area, all experimental groups show bone growth (asterisks) from the border of the defect (b) with immature trabecular conformation surrounded by connective tissue (CT). (**A2**) In the medullary area, for BM + PBMT, BM + HFB and BM + HFB + PBMT, fine bone trabeculae are noted around particles of the biomaterial (BM). (**B**) At 42 days. (**B1**) In the cortical area, increased bone formation in the bone defect border, presence of concentric laminae (inside the blue lined area) in Group 1 (BM + PBMT) and the blood vessel (red arrow) in Group 2 (BM + HFB) are observed. (**B2**) In the medullary area, the bone trabeculae are thicker and more compact with Haversian canals (black arrow) in Group 3 (BM + HFB + PBMT), in relation to the previous period with some biomaterial particles in all groups. (HE; original 100 × magnification; bar = 50 μm).

At 14 days, as seen in Figure 4(A1), all experimental groups presented newly formed bone trabeculae in the medullary region of the defect, permeating the particles of the biomaterial and connective tissue filling the spaces adjacent to the edge of the lesion (Figure 5(A1)), with some integration of the biomaterial to the tissue in normal bone repair and mineralization pattern, besides the absence of inflammatory process. In Group 3 (BM + HFB + PBMT), there was a large amount of connective tissue with vascular shoots filling the medullary spaces (Figure 5(A2)). Overlying the injured cortical region, the groups had a thin layer of periosteum and connective tissue, slight bone neoformation from the edges of the lesion and the central cortical area filled with loose connective tissue.

At 42 days (Figure 4(A2)), the newly formed bone matrix in the medullary region was thicker, with compact conformation and some Havers canals in relation to the previous period, with evidence of a large amount of osteocytes in Group 3 (BM + HFB + PBMT). The collagen fibers were concentrically arranged to the remaining particles of the biomaterial and organized. At the end of the experimental period, the groups still had biomaterial in the cortical (Figure 5(B1)) and medullary (Figure 5(B2)) regions; in the latter region, this was surrounded by newly formed bone tissue. All experimental groups showed the injured cortical tending to close, thus restoring the original bone architecture. Group 3 (BM + HFB + PBMT) showed a more mature lamellar bone formation in the injured cortical region.

### 3.4. Histomorphometric Evaluation

In the histomorphometric evaluation of the volume density of new bone formed, it was found at 14 days that Group 3 (BM + HFB + PBMT) had a mean of 26.4% ± 1.03%, with a statistical difference compared to Group 2 (BM + HFB), which obtained the lowest values (20.0% ± 1.87%); Group 1 (BM + PBMT) presented a mean of 22.2% ± 1.77%, with no significant difference compared to the other groups (Figure 4(B1) Table 1).

At 42 postoperative days, a statistical difference was observed between Groups 3 (BM + HFB + PBMT) and 2 (BM + HFB) (38.2% ± 1.59% and 31.6% ± 1.33%, respectively), with a mean of 33.2% ± 2.18% for Group 1 (BM + PBMT), the latter without statistical difference in relation to the other groups (Figure 4(B2); Table 1).

Analyzing the data in the euthanasia periods (14 and 42 days), within each group, there was a statistical difference between 14 and 42 days for the three groups (Figure 4(B3); Table 1).

## 4. Discussion

The aim of the present study was to verify the influence of PBMT on bone reconstruction of rat tibias by combining two components, heterologous fibrin biopolymer and lyophilized bovine bone matrix. We observed tissue biocompatibility of these scaffolds, in addition to greater maturation of bone tissue in the final period of the experiment. PBMT has shown positive effects on different types of tissue recovery [13,16,31,41], but there are still controversies regarding the appropriate parameters to be used.

The choice of long bones such as the tibia for bone repair is related to its ease of manipulation and access and its similarity to the clinical application in humans, regarding remodeling, repair in the physiology of muscle strength and tension. When dealing with critical defects, larger animals should be used, such as sheep and pigs [31,33,34,36,42]. In addition, biomaterials are used in orthopedic medical surgeries performed to correct bone defects of dimensions that do not spontaneously repair, as well as in patients with osteoporosis or cancer [42,43]. Tissue engineering states that the periosteum of the long bones as an auxiliary element in reconstructive process research, and another factor is the size of defects for biomaterial analysis, with the highest absorption being cited for noncritical defects [44].

The distribution of animals in their respective groups in the present study, as well as the number of specimens (*n*), was based on the principle of the 3 R’s, in which there is a commitment by the world scientific community to follow the Russell–Burch Principles (1959) “reduction, replacement and refinement” in the use of animals that, increasingly, remain active in scientific and academic circles. Therefore, it was decided to not perform groups with defects filled only by clot, autogenous bone [3,17] or Bio-Oss^®^ [5,7,28,45], widely previously published in the literature, including the same methodology used in the present experiment and also from the same research group [39,46,47,48], focusing on only in the originality and aims of the research.

Following the same methodology used in this study, including similar analyzes, Song et al. [49] evaluated a hydrogel based on carboxymethylcellulose (CMC) randomly separating the rats into three groups: CMC/BioC (biphasic calcium phosphate), CMC/BioC/BMP-2 0.1 mg (bone morphogenetic protein-2) and CMC/BioC/BMP-2 0.5 mg, concluding that the hybrid material CMC/BioC/BMP-2 induced greater bone formation than the other tested materials. Likewise, focused only on the tested biomaterials, Kido et al. [36] randomly separated the rats into two groups: Biosilicate group (BG) and poly PLGA Biosilicate group (BG/PLGA), reaching concluding that BG/PLGA showed a faster degradation of the material, accompanied by greater bone formation when compared with BG, after 21 days of implantation.

As observed by the µCT, in this study, all groups tended towards bone neoformation and there was presence of biomaterial particles until the last experimental period. Studies differ between resorption [50] and non-resorption [51] of biomaterial after PBMT, and no study using HFB cited this relationship. It was not possible to perform a quantitative distinction of the newly formed tissue since the xenograft has great similarity with cortical bone, mainly in its radiopacity [52,53], so its application in this analysis has been challenging. In agreement with the literature, we identified in the present study the presence of newly formed bone tissue in the midst of the biomaterial particles, demonstrating active repair progress, and the close relationship between the µCT and histomorphometric analysis as complementary in bone evaluation [53,54].

The groups that used the HFB showed greater visual proximity of particles and integration of components. In addition, its use facilitated the agglutination of the biomaterial particles for insertion in the bone defect, as well as a rapid decrease in bleeding caused by the injury. Initially called as heterologous fibrin sealant (HFS), this bioproduct, derived from snake venom produced by the CEVAP, was used in the recovery of venous ulcers [10,55,56] and as a glue for injured nerves [57]. Next, new experiments showed more advantages of the sealant, such as the ability to act as a scaffold for stem cells [30] or biomaterials [15,16,17,50,58] and as a new medication administration system [9]. Considering that the use of this bioproduct goes beyond its adhesive capabilities, its nomenclature has been reconsidered and has recently been called “fibrin biopolymer”, but there are still not many studies on the osteogenic potential of HFB.

In the present study, the results of the histological analysis showed that the physiological inflammatory process of bone repair was already completed at 14 days in all groups due to the absence of reaction tissue, demonstrating that the association of biomaterial with both HFB and PBMT and both at the same time present biocompatibility characteristics [59,60], corroborating studies associating HFB with autogenous bone and PBMT (830 nm) [17]. Research with 830 nm [18,61] and 808 nm [62] lasers identified the modulatory action of the laser relating to mature bone neoformation with increased osteoblast factor proteins and genes, and the xenograft tested here is widely used in experiments in the literature with applications in animals and humans, with bovine bone being elected with biocompatible properties and good acceptance.

Moreover, in the first analysis period of the study (14 days), the forming bone tissue increased in a centripetal way from the edges of the defect and was characterized by thin and disorganized fibers. However, at the end of the experiment (42 days), newly formed bone tissue was thicker, mature and organized with a physiological process of repair, similar to studies with 830 nm laser [60,63,64] that identified such bone maturation over the analyzed periods. These studies report that especially after 30 days, there is more organization and tissue repair maturation, besides the presence of denser collagen fibers in long bones.

Between Groups 2 (BM + HFB) and 3 (BM + HFB + PBMT), there was a statistical difference in relation to bone percentage in both periods, relating findings compatible with the literature when observing higher bone density [61,65], blood vessels, Havers canal development, maturation and organization of bone tissue, following the principles that the tissue will only respond to biostimulation if the energy is adequate, reaching the minimum limit, and when the energy is excessive, there may be tissue inhibition [22,66].

The PBMT protocol used in the present experiment is due to previous studies (in vivo) on tissue regeneration [8,12,13,16], plus a literature review on the action of PBMT specifically on bone tissue, in which the length of 830 nm was cited as the most used (40.79%) and with positive effects in 98.68% [67]; in another review, satisfactory results were observed between the PBMT and xenograft in 17 of the 18 articles included [23]. The literature points to laser-modulated bone formation with increased bone growth factors in differentiated cells by stimulating matrix secretion, cell proliferation and reduced inflammation [65,68].

The results presented by our study corroborate the results of Iatecola et al. [17], who used autogenous bone graft (ABG) associated with HFB and PBMT. PBMT also showed satisfactory results in helping to reconstruct long bones when associated with biomaterials such as synthetic hydroxyapatite [41], biosilicate^®^ [31], and Celecoxib^®^ [69], in addition to an increase bone mineral density with salmon calcitonin [70].

The biological action of the laser depends on the penetration, propagation, absorption and length of light. The infrared laser is capable of reaching deeper tissues, penetrating about 2 mm into the tissue before losing 37% of energy, so when compared to the red light-length laser (which has 0.5–1 mm triggered energy) [71], it penetrates more with less energy loss, being indicated for bone lesions [19,71,72], affecting mitochondrial stimulation, formation of new blood vessels and proinflammatory and regenerative cytokines [33,73].

PBMT has been shown to be an ally in the process of bone reconstruction; however, the consensus about parameters for the purpose of bone regeneration is still uncertain in the literature, since studies show varieties of protocols, thus obtaining different results [23,74,75] such as differentiation, density and osteogenic proliferation [67,74,76].

## 5. Limitations

The implementation of functional analyses such as biomechanics [33] and immunomarkers [77] for bone cells help to better interpret the process of tissue maturation and may be considered as a limitation of the present research. In addition, a greater number of groups and specimens studied, provided they are ethically approved, can also be considered a limiting factor. We list as future studies the analysis and quantification of collagen fibers [78] and comparison between different concentrations of biopolymer in order to verify its osteogenic characteristics.

## 6. Conclusions

It was concluded that PBMT, through the use of low-level laser, associated with the biocomplex formed by the heterologous fibrin biopolymer (HFB) added to the lyophilized bovine bone matrix (BM), has the potential to aid in the reconstruction process of bone defects in the tibia of rats.

## Figures and Tables

**Table 1:** Table of volume density of new bone formation (%).

	BM + PBMT	BM + HFB	BM + HFB + PBMT
14 days	22.20 ± 1.77 Aab	20.00 ± 1.87 Ab	26.40 ± 1.03 Aa
42 days	33.20 ± 2.18 Bab	31.60 ± 1.33 Bb	38.20 ± 1.59 Ba

Different uppercase letters (comparison in columns, 14 vs. 42 days) indicate a statistically significant difference. Different lowercase letters (line comparison, BM + PBMT vs. BM + HFB vs. BM + HFB + PBMT in each period, 14 or 42 days) indicate a statistically significant difference. Student’s *t* and Tukey’s test, respectively, both at 5% probability.

## Supplementary Materials

The following are available online at https://www.mdpi.com/2218-273X/10/3/383/s1, Figure S1: Representative image of the methodology used to quantify the area of newly formed bone.
