# Supplementary figures and images for: Photobiomodulation Therapy Associated with Heterologous Fibrin Biopolymer and Bovine Bone Matrix Helps to Reconstruct Long Bones
Source: Biomolecules. 2020 Mar 2;10(3):383. doi: 10.3390/biom10030383 (PMC7175234; doi:10.3390/biom10030383)

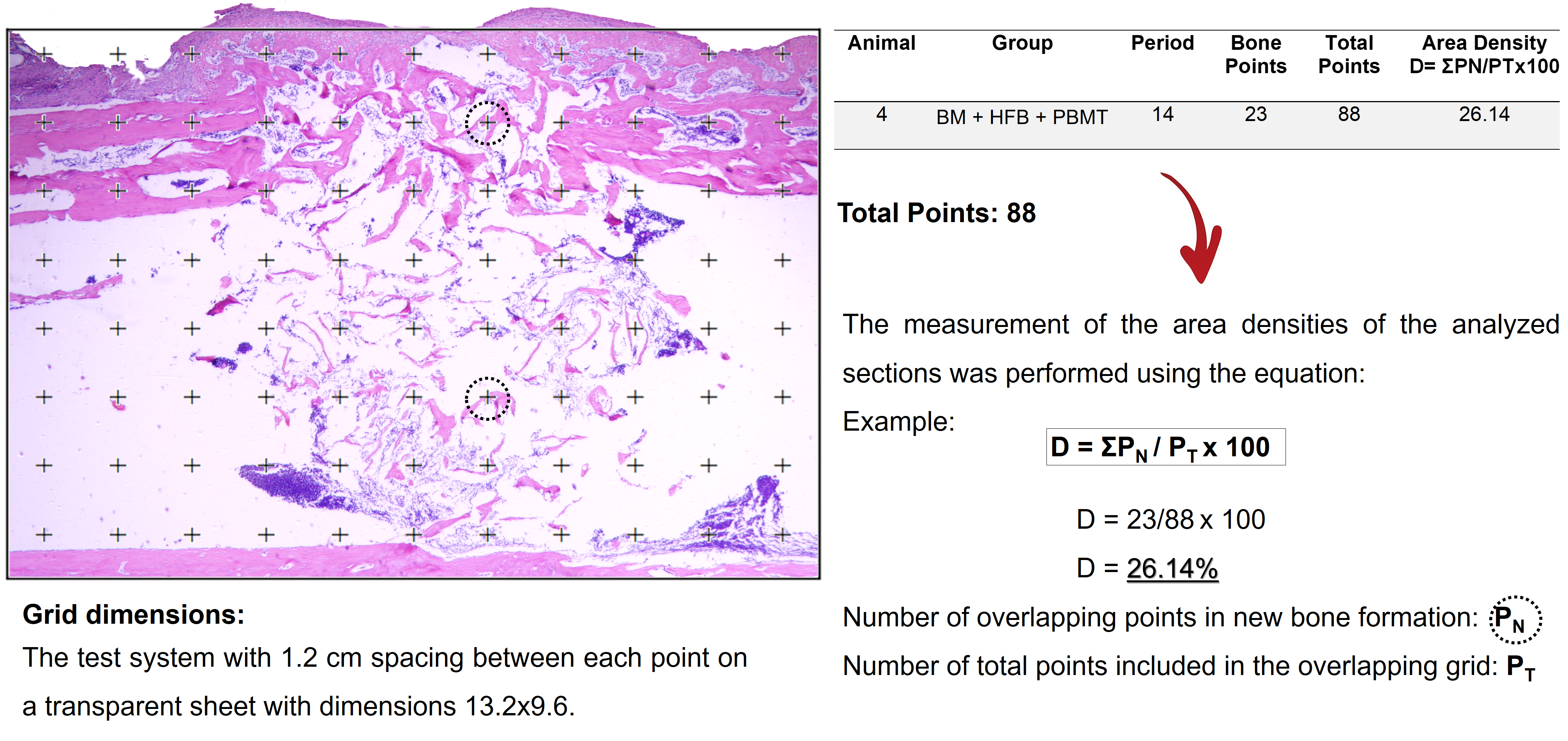

Supplement: Supplementary file 1 [file biomolecules-10-00383-s001.tif]
